# Molecular Mechanisms of Periodontal Disease

**DOI:** 10.3390/ijms22020930

**Published:** 2021-01-19

**Authors:** Mikihito Kajiya, Hidemi Kurihara

**Affiliations:** Department of Periodontal Medicine, Graduate School of Biomedical and Health Sciences, Hiroshima University, 1-2-3 Kasumi, Minami-Ku, Hiroshima 734-8553, Japan; hkuri@hiroshima-u.ac.jp

Periodontal disease, one of the most prevalent human infectious diseases, is characterized by chronic inflammatory tissue destruction of the alveolar bone and the connective tissues supporting the tooth. Such pathological inflammation is caused by the host innate and adaptive immune response to a constellation of periodontal biofilm-related multiple microorganisms. The current knowledge of the pathophysiology of periodontal diseases is involves identifying keystone bacteria in the biofilm, the host immune response (including genetic factors), and environmental stressors. Based on these accumulated lines of evidence, novel diagnosis, preventive, or therapeutic remedies are under development or envisioned. Briefly, the sum of all these scientific endeavors will deepen the understanding of more detailed molecular mechanisms of periodontitis, thereby contributing to advances in therapeutic strategies for periodontitis. Thus, this Special Issue, entitled “Molecular Mechanisms of Periodontal Disease”, ten articles, three systematic, and six literature review papers are presented, highlighting recent advances in “the relation between keystone bacteria and immune system”, “the role of bacterial/host metabolites”, “the mechanism of osteoclastogenesis”, “the genetics, epigenetics, and environmental factors”, “the potential liquid biopsy for periodontitis”, and “the novel strategy for the regenerative therapy”.

As described above, periodontitis is elicited by the host immune response against periodontal pathogenic microorganisms [1]. Recent advanced studies proposed that periodontitis is caused mainly by dysbiosis of the bacterial community, rather than an infection by individual bacteria. This new concept pushed the researchers to identify the keystone bacteria and understand how the host immune system responds to the dysbiosis [1]. One of the vital recognition systems for microbial dysbiotic change is toll-like receptors (TLR). Moonen et al. reported that activation of TLR2 in gingival fibroblasts or monocytes is responsible for the T cell proliferation [2]. Consistent with this study, it has long been demonstrated that the TLR-mediated innate immune system by pathogen-associated molecular patterns (PAMPs) activates T and B cells’ immune responses to produce inflammatory cytokines and elicits osteolytic pathways, promoting periodontal inflammation and bone resorption. However, interestingly, recent studies demonstrated that immune regulatory T and B cells are also induced by the activated innate immune system, suggesting that several adaptive immune systems play a protective role in the context of periodontitis. The reader can refer to the excellent review by Gu et al. for the distribution of the T/B lymphocytes’ phenotype [3]. 

It is also well accepted that not only PAMPs but also several metabolites secreted from bacteria or host cells are associated with the progress of periodontal disease. For instance, short-chain fatty acids (SCFAs), including acetate, propionate, and butyrate, which are Gram-negative bacterial metabolites, are frequently detected in the periodontal pocket of periodontitis patients. Magrin et al. revealed that, among the SCFAs, butylate might exacerbate the gingival epithelium inflammatory response in their in vitro study [4] and systematic review [5]. On the other hand, the lipids secreted from dying peripheral blood mononuclear cells activate macrophages’ inflammatory cytokine production [6]. 

Receptor activator of nuclear factor-kappa B ligand (RANKL) is a master cytokine of osteoclast differentiation and function. Although it is still controversial, osteoblasts or stromal cells are thought to be the cellular source of RANKL in the context of periodontitis. Thus, much scientific attention has been paid to the study of osteoblasts and osteoclasts. Kelder et al. reported that osteoblastic cells isolated from long bone showed more significant osteogenic activity than those of alveolar bone [7]. As the novel molecular mechanisms underlying RANKL induction in osteoblasts, the significant role of LAMP-2, which is involved in the fusion of lysosomes with phagosomes, was discovered by Jansen et al. [8]. On the other hand, to investigate the role of pro-inflammatory cytokines in RANKL-induced osteoclastogenesis, Ascone et al. employed IL-1 receptor antagonist-deficient (IL1rn-/-) mice. As a result, they demonstrated that the disruption of the IL-1 inhibitor increased the number of osteoclast precursors in the bone marrow, which resulted in increased bone loss [9]. 

The genetic background of the host immune system seems to be associated with the onset of periodontitis caused by pathogenic bacteria. A case–control study by Borilova et al. indicated one possibility that the polymorphism IL-10-1087G/C (rs1800896) and specific IL-10 haplotypes may play a role in the development of periodontal disease [10]. Moreover, a systematic review by Khouly et al. implied that epigenetic modification, which changes gene expression by DNA methylation but not the DNA sequence, may also be involved in the onset of periodontal disease [11]. In addition, the other environmental factors, including senescence and stress, are also responsible for the progress of periodontitis. Based on the emerging evidence, in their review article, Aquino-Martinez et al. proposed a new scientific model that senescent cells, which receive oxidative stress and subsequent DNA damage, may aggravate the innate immune reaction against pathogens [12]. Lopes et al. indicated that chronic stress-induced blood oxidative stress exacerbates periodontal bone resorption using the rat physical restraint model [13]. 

Given its intrinsic nature, gingival crevicular fluid (GCF) and saliva can be an attractive liquid biopsy for periodontitis. In combination with the advanced technology for proteomics analysis, Preianò et al. proposed a future reliable diagnostic system using GCF [14]. Han et al. reported that the size exclusion chromatography method is appropriate for salivary small extracellular vesicle isolation, which harbors DNA methylation genes and microRNAs, such as miR-146a-5p, reflecting the physiological condition [15,16]. Interestingly, the systematic review by Asa’ad et al. indicated that miR-146a could be utilized as a fluid biomarker in periodontitis patients [17]. Future studies establishing the applicable liquid biopsy for periodontitis should be helpful for all clinicians and patients. 

To date, several regenerative therapies using stem cells, growth factors, and biomaterials have been tested with the aim of successful periodontal tissue regeneration. In any case, emerging evidence implies that both osteogenesis and angiogenesis are indispensable for successful tissue regeneration. This relationship between osteogenesis and angiogenesis is well documented in the review article by Diomede et al. [18]. Recently, periodontal therapy using laser irradiation, so-called “periodontal phototherapy”, has attracted much dental attention. Importantly, since laser irradiation can facilitate vascular endothelial cell proliferation and osteoblast, periodontal ligament cell, and cementoblast differentiation, periodontal phototherapy might be applicable as a novel periodontal tissue regenerative therapy. This promising perspective is reviewed by Ohsugi et al. [19]. 

For decades, scientific endeavors in periodontology have made several paradigm shifts, including keystone bacteria-mediated dysbiosis, immune regulatory T/B cells, RANKL-producing stromal cells, and genetic and epigenetic inflammatory responses. Moreover, following these discoveries, novel promising therapies are under development. In this Special Issue, nineteen cutting edge scientific papers in this study field are introduced. We are confident that scientists, including all the authors involved in this Special Issue, will continue to provide unexpected discoveries that will lead us to understand the molecular mechanisms of periodontal disease. This, in turn, will encourage us to develop novel promising preventive and therapeutic strategies for periodontitis patients.
